# STAGEs: A web-based tool that integrates data visualization and pathway enrichment analysis for gene expression studies

**DOI:** 10.1038/s41598-023-34163-2

**Published:** 2023-05-02

**Authors:** Clara W. T. Koh, Justin S. G. Ooi, Eugenia Ziying Ong, Kuan Rong Chan

**Affiliations:** 1 Duke-NUS Medical School, Programme in Emerging Infectious Diseases, 8 College Road, Singapore, 169857 Singapore; 2 Viral Research and Experimental Medicine Center @ SingHealth Duke-NUS (ViREMiCS), Singapore, Singapore

**Keywords:** Data processing, Software, Gene expression, Software

## Abstract

Gene expression profiling has helped tremendously in the understanding of biological processes and diseases. However, interpreting processed data to gain insights into biological mechanisms remain challenging, especially to the non-bioinformaticians, as many of these data visualization and pathway analysis tools require extensive data formatting. To circumvent these challenges, we developed STAGEs (**S**tatic and **T**emporal **A**nalysis of **G**ene **E**xpression **s**tudies) that provides an interactive visualisation of omics analysis outputs. Users can directly upload data created from Excel spreadsheets and use STAGEs to render volcano plots, differentially expressed genes stacked bar charts, pathway enrichment analysis by Enrichr and Gene Set Enrichment Analysis (GSEA) against established pathway databases or customized gene sets, clustergrams and correlation matrices. Moreover, STAGEs takes care of Excel gene to date misconversions, ensuring that every gene is considered for pathway analysis. Output data tables and graphs can be exported, and users can easily customize individual graphs using widgets such as sliders, drop-down menus, text boxes and radio buttons. Collectively, STAGEs is an integrative platform for data analysis, data visualisation and pathway analysis, and is freely available at https://kuanrongchan-stages-stages-vpgh46.streamlitapp.com/. In addition, developers can customise or modify the web tool locally based on our existing codes, which is publicly available at https://github.com/kuanrongchan/STAGES.

## Introduction

Gene expression profiling has emerged as a powerful tool for biomedical research. With high-throughput microarray and RNA sequencing, it is now possible to measure gene expression rapidly and cost-effectively in cells and tissues across multiple time-points, leading to an exponential increase in publicly available transcriptomic datasets in the recent years. Compared to a single static snapshot, adding a third dimension of time offers deeper insights into the biological mechanisms involved, as tracking temporal changes can evaluate not only when transcriptomic changes matter, but also the duration of transcriptional responses that are influenced by experimental conditions. Indeed, we and others have demonstrated that day 1 host transcriptional responses to the YF17D vaccine was associated with adverse events^1^ but the correlates of vaccine immunogenicity were more apparent at days 3–7^2,3^. In another study, by daily tracking of severe and mild COVID-19 patients, we ascertained that neutrophil signatures but not interferon signaling are associated with respiratory nadir^4^. Overall, these studies highlight the importance of time-series gene expression profiles to better understand how trajectory of gene expression changes impact biological phenomena.

The current challenge no longer lies in obtaining gene expression profile and data pre-processing, as specialised software and tools such as Partek Genomics Suite, Limma package and Transcriptomic Analysis Console can handle these processes efficiently^5^. However, interpreting the processed results to gain insights into biological mechanisms remain challenging, as pathway analysis tools are not centralised and typically require extensive data formatting to utilise these tools. The development of pathway analysis tools is an active research field, and algorithms implemented in Enrichr and Gene Set Enrichment Analysis (GSEA) are efficient tools for interpreting gene expression data^6–9^. However, implementing these tools can be laborious and error-prone when many samples and comparisons are involved. Although programming frameworks like Python, R and Bioconductor libraries can facilitate omics data analysis, these bioinformatic tools may be challenging for users without coding or programming background.

To overcome these issues, we present STAGEs (Static and Temporal Analysis of Gene Expression Studies), which is a web-based and high-throughput analysis pipeline with an intuitive user interface that allows systematic characterisation of static and temporal transcriptomic data. Besides comparisons between time-points, different treatment conditions can also be compared, allowing for multiple comparison analyses. STAGEs integrate the use of various data visualization tools, as well as pathway enrichment analyses to allow users to explore transcriptomics data tailored towards their own needs. Output data tables and graphs are interactive and can be customized using widgets such as sliders, drop-down menus, text boxes and radio buttons located at the side bar. Finally, as our web browser is created by Streamlit, developers can conveniently amend the Python codes to include customized gene set files (https://github.com/kuanrongchan/STAGES) for a deeper characterization of the genes and pathways that are differentially modulated.

## Overview of STAGEs

**Figure 1:**
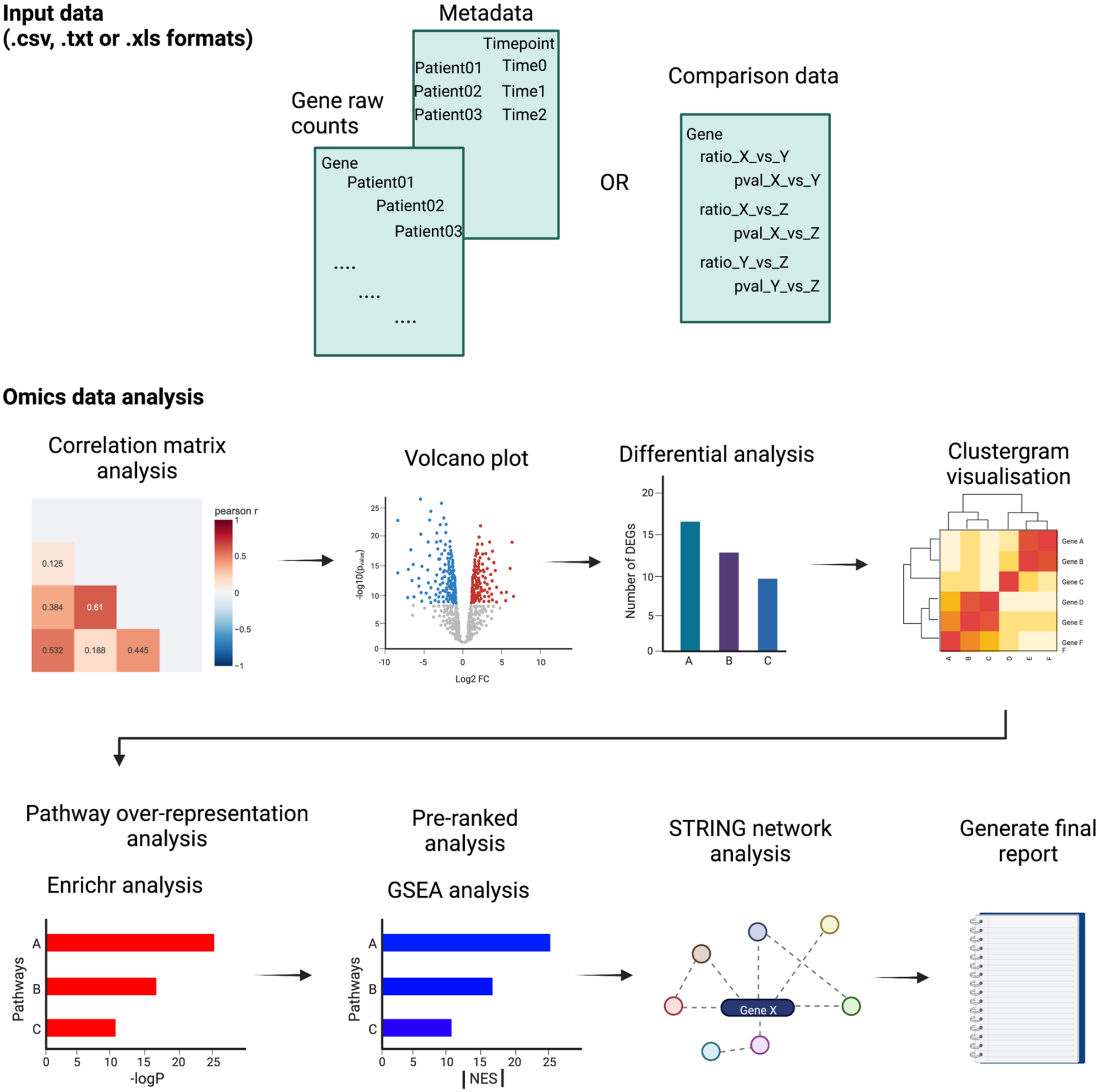
Schematic view of STAGEs platform. Raw counts or comparison data files (in csv, txt or xls formats) can be uploaded to STAGEs for omics data analysis. The data will then be analysed sequentially, to generate correlation matrices, volcano plots, differential analysis plots, clustergrams, pathway enrichment charts, and protein–protein interaction networks. Results are then saved in the final report, which can be downloaded.

STAGEs is an interactive web app built using Streamlit (https://www.streamlit.io), and the running instance of the online app can be accessed via the website (https://kuanrongchan-stages-stages-vpgh46.streamlitapp.com/). The app can also run locally using the instructions detailed in GitHub (https://github.com/kuanrongchan/STAGES). Users can directly upload data from Excel spreadsheets, csv or txt files containing ratio and p-values into STAGEs, where STAGEs will first auto-correct for any Excel gene-to-date conversion errors with Gene Updater^10^, ensuring that every gene will be considered for pathway analysis. Alternatively, raw counts from RNAseq or log2 counts from microarray data can be uploaded, and the webtool will then tabulate the fold-change and p-values for downstream analysis (Fig. 1). Thereafter, users can select the apps at the side bars to render correlation matrices, volcano plots, differentially expressed genes as stacked bar charts, clustergrams, and pathway enrichment analysis by Enrichr and Gene Set Enrichment Analysis (GSEA) (Fig. 1). The output is a personalised data report that displays the results from data analysis, where users can manipulate parameters using widgets such as sliders, drop-down menus, text boxes and radio buttons located at the side-bar. When parameter settings are changed, the results are automatically re-calculated and updated in the dashboard real-time. The output graphs are visualised with either the Python graphic libraries Matplotlib or Plotly, the latter allows generation of interactive graphs that can show data upon mouseover. The uploaded data and output of the analyses are not stored anywhere, ensuring the safety and security of the data.

### Data input

STAGEs can work on Google Chrome, Firefox and Microsoft Edge, and on MacOS, Windows and Linux. The user interface of STAGEs starts with a file uploader that enables users to upload Excel, csv or txt comparison file(s). To upload a comparison file, the file should contain annotation labels (e.g. gene names) on the first column, ratio values (relative transcript expression comparing control vs baseline) and the corresponding p-values. For the web tool to recognise the ratio and p-value columns, users will need to label as ratio_X_vs_Y and pval_X_vs_Y respectively, where X and Y are the comparison variables separated by underscore. The X and Y variables can be time-point comparisons (e.g. ratio_day1_vs_day0, ratio_hr6_vs_0) or experimental-control comparisons (e.g. ratio_drugA_vs_placebo, ratio_virus_vs_ctrl). Moreover, multiple pairwise comparisons can be performed simultaneously by adding their respective ratio and p-value columns to the dataframe (e.g. ratio_A_vs_Y, pval_A_vs_Y, ratio_B_vs_Y, pval_B_vs_Y). Finally, STAGEs can also perform multiple comparisons for time-course studies by allowing users to upload multiple files. However, the time-points and labelling must be consistent across the different experimental conditions for multiple file comparisons. For users interested to explore all features within STAGEs, a demo dataset showing the gene transcript expression levels in seronegative subjects after MERCK Ad5/HIV vaccination^11^ is pre-loaded.

Alternatively, users can generate the comparison file within STAGES by uploading the gene raw counts from RNAseq or log2-normalised counts from microarray datasets into STAGEs for pre-processing. The metadata containing the attributes of the samples should also be included so that the comparison file can be generated. Users can then select the variables and statistical tests to use for comparisons within STAGEs. Similarly, demo datasets are provided for users to explore the web tool and workflows.

After uploading the comparison data file, users can inspect the data by clicking on the checkbox at the side bar. By clicking on the header columns, users can sort numeric values either in ascending or descending order to ensure that the correct dataset is successfully uploaded into the STAGEs web tool. We have also incorporated the Gene Updater into STAGEs at backend^10^, so that the old gene names and date terms will be auto-converted to the new gene names as recommended by the HUGO Gene Nomenclature Committee (HGNC).

### STAGEs output

STAGEs allow users to visualise correlation matrices, volcano plots, DEG stacked bar charts, cumulative distribution functions, clustergrams, pathway enrichment analysis from Enrichr and GSEA and protein–protein interaction networks. Users will be prompted to perform the data analysis in a sequential manner, and users can familiarise themselves with the workflow using the demo dataset that is pre-loaded within the web tool. STAGEs documentation is also provided at the front page of the web tool and GitHub (https://github.com/kuanrongchan/STAGES) for users to understand the features and capabilities of the web tool. After analysis, users can download the results of the DEG and pathway analyses as Excel files, and the output charts collectively as a report format.

### Correlation matrix

**Figure 2:**
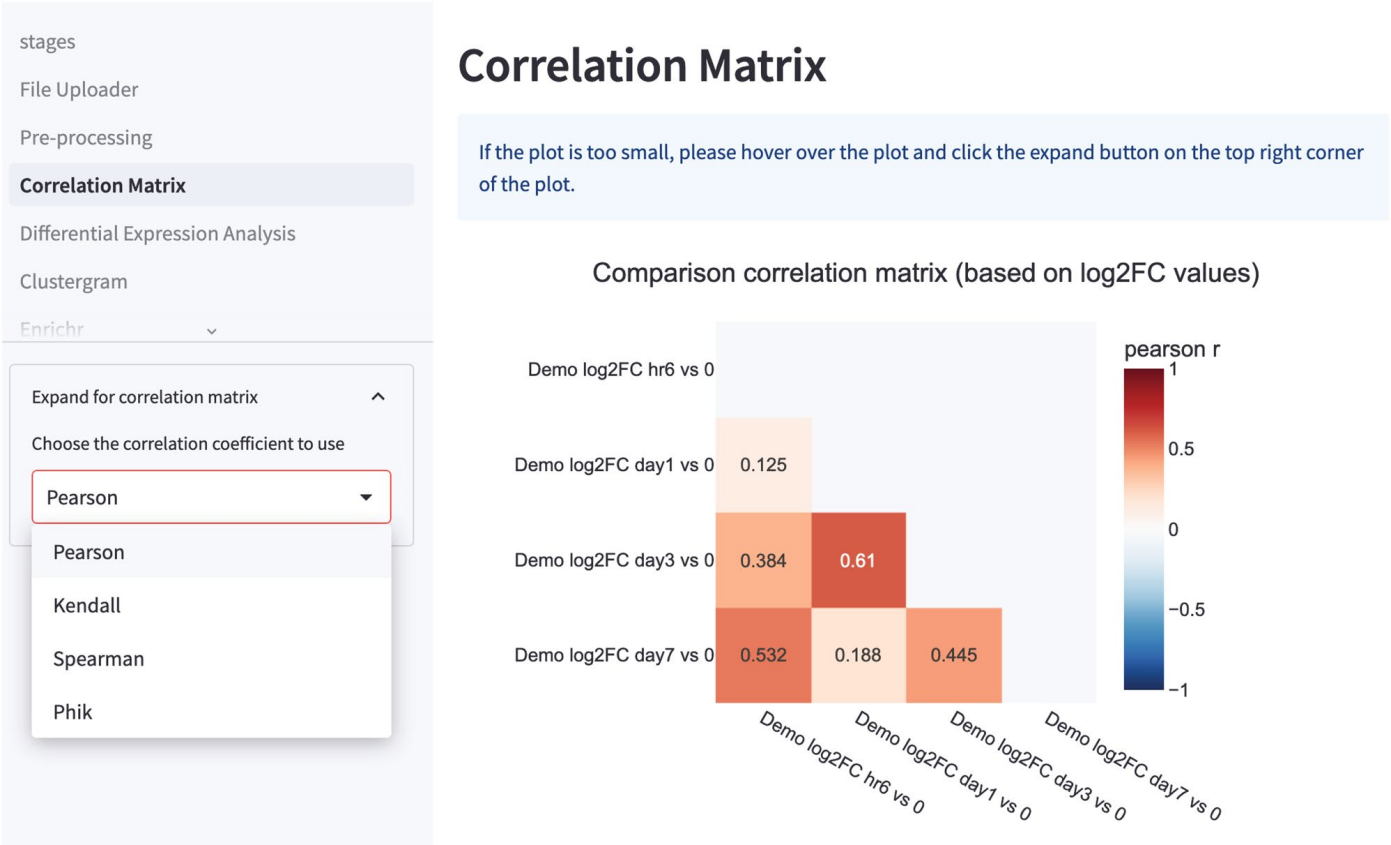
Correlation matrix rendered by STAGEs. Correlation matrix between different pairwise comparisons can be rendered by STAGEs to ascertain if the expression changes are correlated between different experimental conditions. Users can select to perform Pearson, Spearman, Kendall and Phik correlation for their analysis.

The first graph rendered is the correlation matrix, to compare relatedness in host transcriptomics responses between the different experimental conditions. STAGEs converts the ratio values to log2-transformed fold change values at backend, and the correlation matrix is generated by performing pairwise correlations of the log2-transformed fold changes between the different experimental conditions. Depending on the user’s preference, users can perform either the Pearson, Spearman, Kendall or Phik correlation for the pairwise comparisons. The output is a correlation matrix showing the pairwise correlation coefficient values (Fig. 2).

### Differential expression analysis

**Figure 3:**
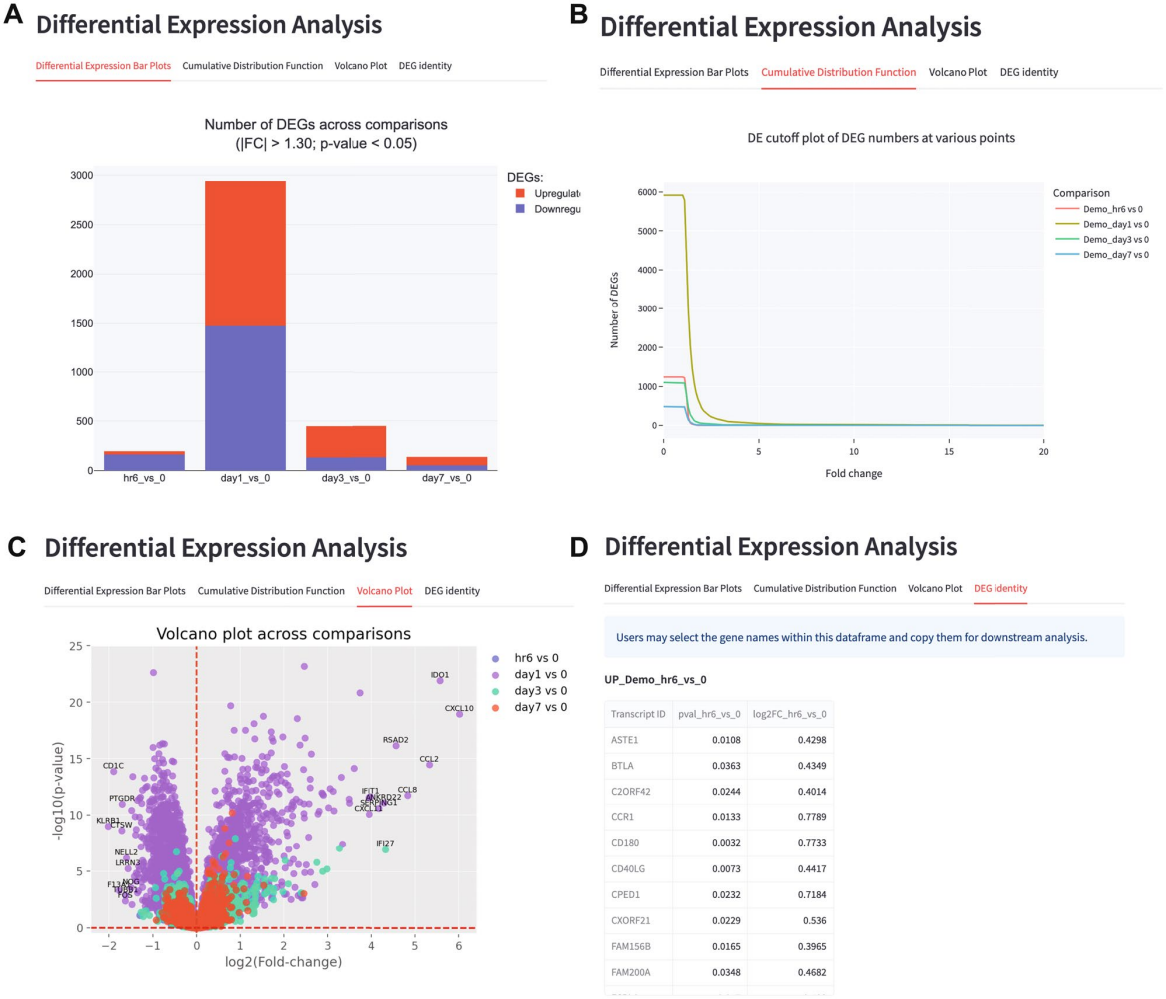
Differential expression analysis charts rendered by STAGEs. (**A**) Stack bar charts rendered by STAGEs, which displays the number of upregulated and downregulated differentially expressed genes. The threshold parameters such as p-value and fold-change values can be adjusted using the sliders located at the left of the side bars. In this case, we used the default threshold, which is fold-change > 1.30 and p-value < 0.05. Results based on demo dataset within STAGEs. (**B**) Cumulative distribution function graph displaying the number of DEGs based on user assigned p-value cutoff. Attributes of the data points can be rendered upon mouse-over. (**C**) Volcano plot based on demo dataset within STAGEs, where ratios and p-values are automatically converted to log2(fold change) values and -log10(p-values) respectively. Settings can be adjusted using sliders located at the side bar of the app. Interactive volcano plots can be rendered by clicking on the checkbox located at the side bar. (**D**) Identity of DEGs after setting the fold-change and p-value cutoffs in A. Data can be downloaded as an xlsx file for further analysis.

Next, users will be able to display the charts related to differential expression analysis. First, to render the DEGs stacked bar chart, users can define the fold-change and p-value cutoffs at the side-bar, and the corresponding stacked bar chart showing the number of upregulated and downregulated DEGs will then be updated in real-time on the STAGEs dashboard (Fig. 3A). This interactive feature allows users to optimise the fold-change and p-value cutoffs, to increase the likelihood of yielding meaningful biological insights from downstream pathway enrichment analyses^13^. In addition, users can visualise the number of DEGs based on the different cutoffs by rendering the cumulative distribution function app (Fig. 3B). The mouse-over feature within the app allows users to quickly identify the number of DEGs based on a specified cut-off.

To visualise the distribution of fold change and p-values of all data points, users can render the volcano plot. Python commands are executed at backend, where the ratios and p-values are converted to log2-transformed fold-change values and negative log10-transformed p-values respectively. These values are then displayed as a scatterplot using the Matplotlib and Plotly visualisation library. As the top differentially expressed genes are also ideal targets for validation and demonstrated to be most reproducible across multiple platforms^12^, the top 10 most upregulated and downregulated are annotated in the volcano plots (Fig. 3C). Users can use the sliders at the side bar to customize the range of log2-fold change and p-values to plot, as well as configure the range of x and y-axis values. Users can also select the option to plot an interactive volcano plot using the Plotly library. This option allows users to display the gene and data point attributes when the mouse cursor is hovered over each of the individual data points. If multiple comparisons are indicated in the uploaded dataset, the volcano plots of each comparison will be overlaid, allowing users to directly compare distribution of volcano plots between the different experimental conditions or timepoints (Fig. 3C). After setting the appropriate fold-change and p-value cutoffs, the identity of the DEGs, together with the respective log2 fold-change and p-values will also be extracted into tables, which can be downloaded and exported as an Excel spreadsheet (Fig. 3D).

### Clustergram

**Figure 4:**
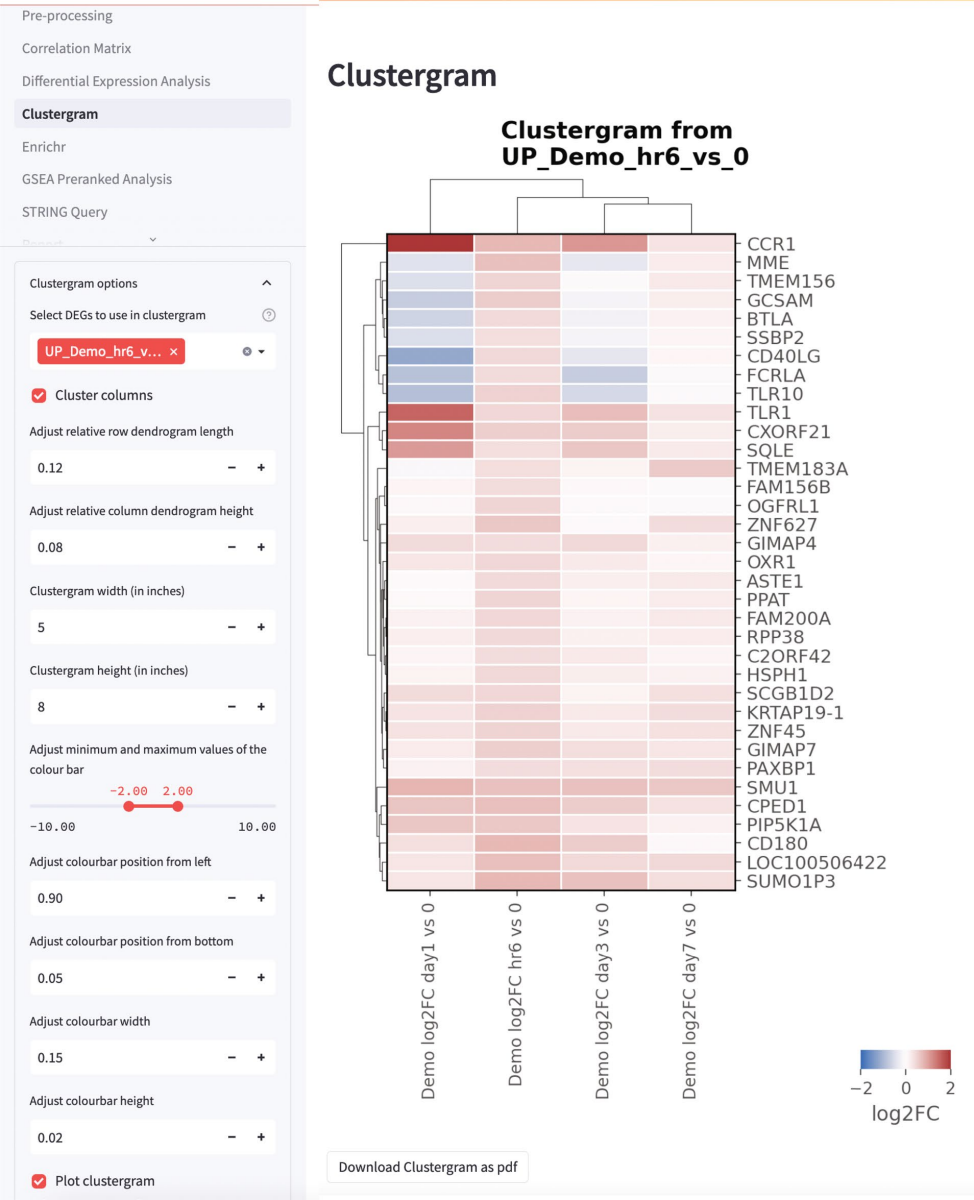
Clustergram rendered by STAGEs. DEGs can be used as input to visualize the magnitude changes and identify unique clusters by unsupervised clustering. Settings for log2(fold-change) magnitude and clustergram dimensions can be tailored at the side bars. Besides DEGs, users can also input a gene list to be used for clustergram analysis. Data based on demo dataset.

After DEG analysis, users can input the upregulated or downregulated DEGs into the clustergram app to visualize the changes in gene expression profile between the different experimental conditions. Alternatively, users can directly copy-and-paste the genes from DEG or pathway analyses to plot clustergrams based on the user-defined gene list. Within the app, users can then customize the range of log2-transformed fold-changes to be plotted, click the option to cluster or not cluster columns, and adjust the height and width settings of the clustergram located at the sidebar (Fig. 4).

### Pathway analysis output with Enrichr analysis

The DEGs determined from the DEG analysis can be subsequently queried against curated pathway databases such as Gene Ontology (GO)^14^, Reactome^15^, KEGG^16^ and HumanCyc^17^ to understand the role of DEGs in biological processes, functions, metabolism and their cell localisation. Users can select the upregulated or downregulated DEGs as their gene input list to query against GO Biological Processes, GO Molecular Function, GO Cellular Component, Reactome, KEGG and HumanCyc databases. In addition, we have added the blood transcription modules (BTMs) curated by Li et al., which is a curated database comprising of an integrated large-scale network of publicly available human blood transcriptomes^18^. Querying against the BTM database allows users to evaluate if their DEGs are related to immune cell subset changes and functions. To demonstrate that the app can also be customised by bioinformaticians to work on in-house gene sets, we curated peak vaccine transcriptomics responses from different vaccines (Supplementary Table 1) and termed this database as Vaccinomics database, for users to ascertain if their DEGs are similar to host response changes elicited by various vaccine types. The gene set information on Vaccinomics database and the .gmt file is available in the GitHub repository (https://github.com/kuanrongchan/STAGES). Lastly, in addition to the pathway databases provided by STAGEs, users can also upload their preferred gene set file within STAGEs for pathway enrichment analysis to query against any other databases.

**Figure 5:**
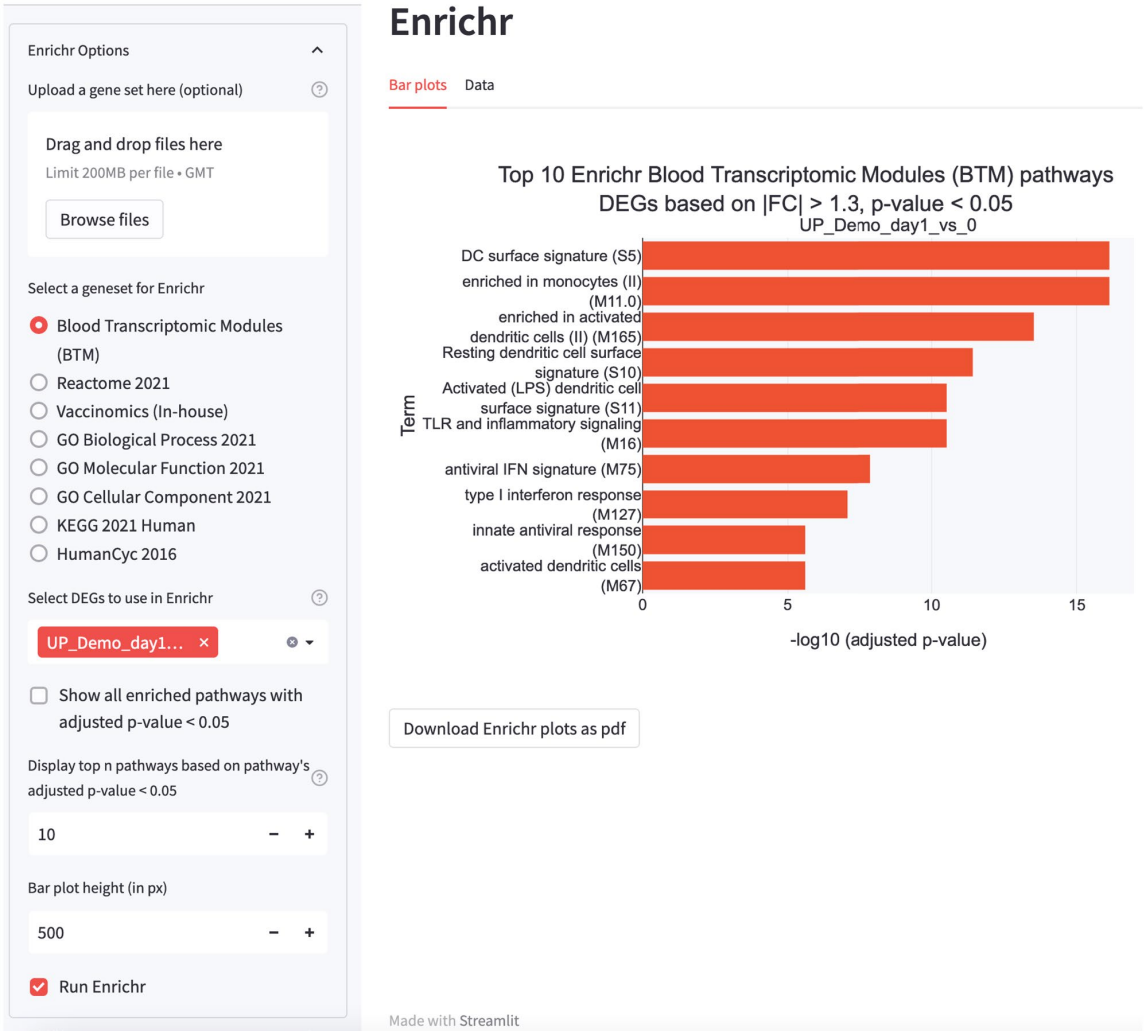
Over-representation analysis output by STAGEs. Horizontal bar charts rendered by STAGEs, which displays the top 10 significant pathways based on the cutoff determined in the DEG analysis in Fig. 3A. In this case study, the upregulated DEGs at day 1 of the demo dataset were queried against the Blood Transcriptomic Modules. Users can query against other databases such as Reactome, Gene Ontology, KEGG, HumanCyc and Vaccinomics within STAGEs. Alternatively, users can also upload their gene sets to query against the other databases that are not provided within STAGEs.

After selecting the pathway database to query against, the raw gene set analysis files showing the extent of overlap between DEGs and pathway database, p-values, adjusted p-values and the identity of DEGs involved in the respective pathways are displayed, and can be downloaded by users for further downstream analysis. By default, the top 10 enriched pathways with adjusted p-values < 0.05 are presented as horizontal bar graphs, in descending order of significance (Fig. 5). Users can also change the settings to display all pathways with adjusted p-values < 0.05, or select the number of top pathways to be presented. Finally, the results for the Enrichr analysis can be downloaded in an Excel format for further downstream analysis.

### GSEA preranked output

Biological data may be noisy and heterogeneous. Thus, modest transcriptomics differences may not be effectively captured with Enrichr as no genes may meet the threshold for statistical significance. Moreover, transcriptomics responses measured by microarray or RNAseq with poor read depth may add to the data noise, causing only few DEGs detected. An alternative is to use the Gene set enrichment analysis (GSEA), which relies on ranking of ratio values to determine leading edge genes responsible for pathway enrichment^8^. However, to implement GSEA, users will typically have to reformat their dataset and download the appropriate database files, which can be tedious and time consuming. With STAGEs, users can perform GSEA prerank analysis without reformatting. The Python codes at backend sorts and ranks the ratio values to identify the pathways which are most significantly enriched. The Reactome, BTM and vaccinomics databases are made available for GSEA prerank analysis in STAGEs. Users may also or upload their gene sets of interest or modify the codes to include other pathway databases if required.

**Figure 6:**
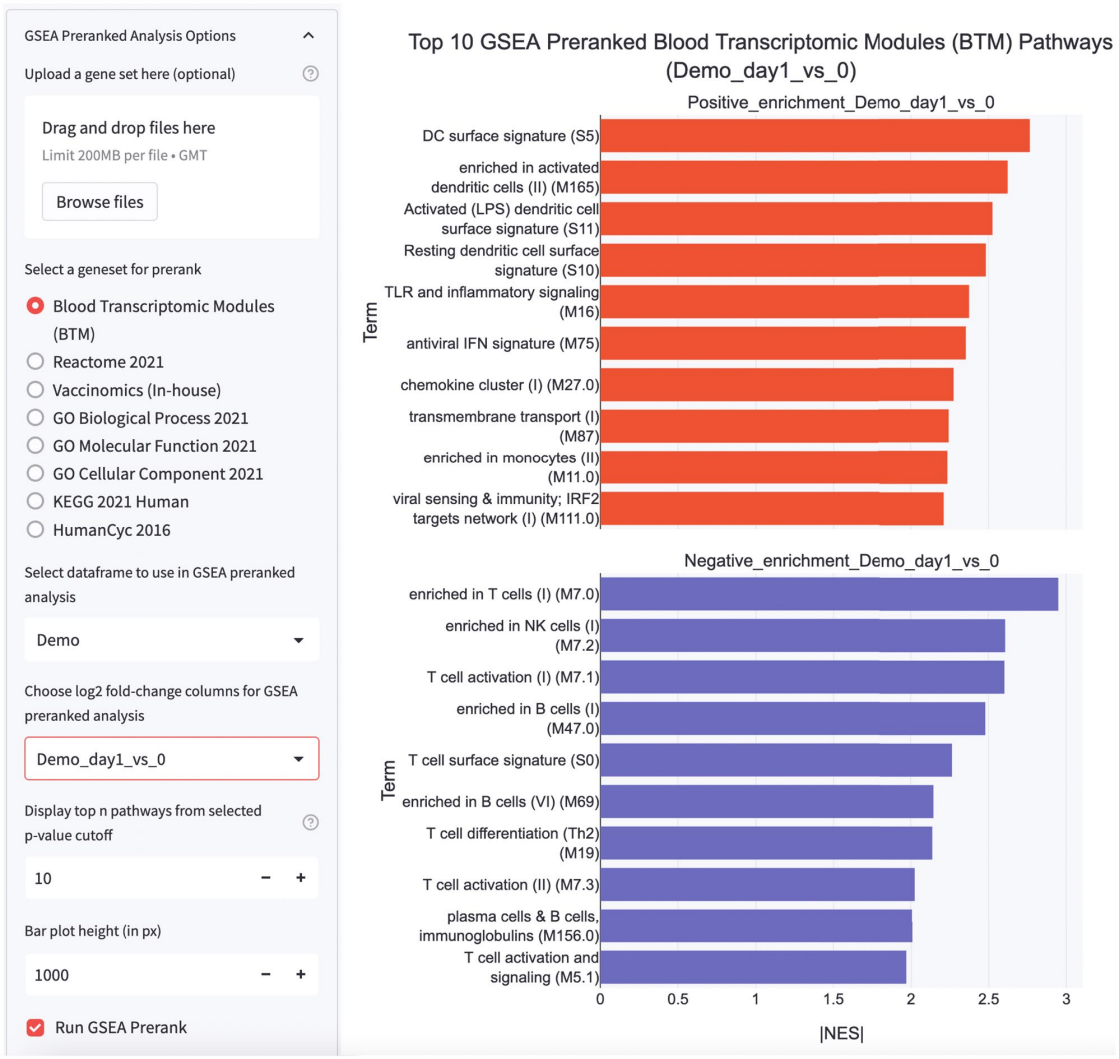
Gene set enrichment analysis output by STAGEs. Horizontal bar charts rendered by STAGEs displaying the top 10 positively enriched (red) and negatively enriched pathways (FDR < 0.05) (blue) based on ratios at day 1 of the demo dataset. In this case study, the ratio values were ranked and GSEA was performed against the Blood Transcriptomic Modules (BTM) database. Users can also upload their gene sets to query against the other databases that are not provided within STAGEs.

After selecting the database to query against, the top 10 positively and negatively enriched pathways are plotted on horizontal bar graphs, in descending order of significance. Users may select to display the pathways with adjusted p-values < 0.05. The raw table file showing the leading-edge genes, normalized enrichment scores (NES) and false-discovery rate (FDR) for the respective pathways can also be downloaded (Fig. 6).

### STRING network analysis

**Figure 7:**
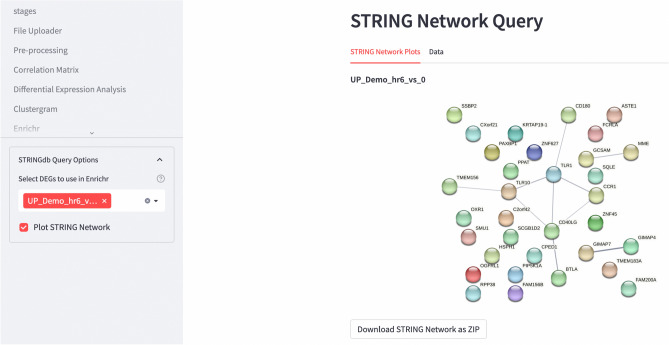
Pathway clustergram and protein–protein interaction network rendered by STAGEs. DEGs can be used as input to perform STRING analysis, to ascertain if the genes act individually, or interact with each other to function a protein complex.

To visualise the protein–protein interaction networks in DEGs, users can render the STRING^19^ query app and select the upregulated or downregulated DEGs as input. Alternatively, users can directly copy-and-paste the genes from pathway analyses as input to understand the protein–protein interaction network in the enriched pathways (Fig. 7). The interaction network serves as an exploratory feature to determine if the gene expression changes are changed individually or as a protein complex. To make finer adjustments to the interaction networks, users can also visit the STRING-DB (https://string-db.org/) for further analysis.

### Comparison with other omics web-tools

Next, we compared STAGEs with other existing web-tools^20–22^. As detailed in Table 1, STAGEs provide more analysis options for correlation and clustering analysis. In addition, multiple pair-wise comparisons can be easily performed with STAGEs, which can be useful for time-point analysis. Finally, STAGEs allow users to upload their own gene sets for pathway enrichment analysis, so users do not have to be restricted to only the databases provided in the webtool (Table 1). However, we do acknowledge that our webtool does not perform batch correction, dimension reduction analysis and machine learning, and some of these features are available in the other existing web-tools.

**Table 1:** Comparison of STAGEs with the other existing webtools.

	GeneCloudOmics	GENAVi	iDEP	STAGEs
Correlation analysis	Pearson, Spearman	No	No	Pearson, Spearman, Kendall, Phik
Volcano plot	Yes	Yes	Yes	Yes, allows multiple pairwise comparisons
DE analysis	Yes	Yes	Yes	Yes
Pathway analysis methods	ORA, GSEA	ORA, GSEA	GAGE, GSEA, PGSEA, ReactomePA	ORA, GSEA
Pathway analysis gene sets	GO, KEGG, MSigDB, WikiPathways	GO, KEGG, MSigDB, WikiPathways, Disease Ontology	GO, KEGG, TF:Target, miRNA.Targets, MSigDB, PPI, Drug, Cancer pathways	GO, KEGG, HumanCyc, Reactome, Blood Transcriptomic Modules Users can upload their own gene sets for pathway analysis
Gene/Sample clustering	Yes	Yes	Yes	Yes. Users can also plot customised heatmaps using enriched genes or leading-edge genes
Protein–protein interaction	Yes	No	Yes	Yes

## Discussion

Presently, plotting volcano plots, DEG analysis, pathway enrichment analysis, protein–protein interaction networks, hierarchical clustering and correlation matrices require copying and formatting of data from one application to another, which can be cumbersome, error-prone and laborious. Indeed, web-based applications and tools such as Appyters^23^ have been designed to allow experimental biologists without coding background to execute bioinformatics workflows for data analysis. However, the main advantage that STAGEs provide is that all of omics workflows, from volcano plots to pathway enrichment analysis to protein–protein interaction networks can be performed within one single app, allowing users to systematically analyse transcriptomic profiles across multiple time-points and experimental conditions. Moreover, by incorporating the Gene Updater framework within STAGEs, users need not worry about missing any genes due to Excel’s gene to date auto-conversion^10^. After familiarization with the web tool, most users can complete their gene expression data analysis within ~ 30 min, highlighting its accessibility and ease-of-use. As the app is designed to analyse the data in a step-wise manner, users can systematically analyse transcriptomics data starting from a broad perspective to the specific biological pathways and networks.

From a developer’s viewpoint, the advantage of using Streamlit is it is easy to use, customize, manage and deploy. Web developers can easily edit the codes to add or remove web tool functionalities. They can also select their preferred database of interest or create their own customized gene sets for pathway analysis. Hence, we envisage that the utility of STAGEs is not only confined to analyzing gene expression data but can be applied more broadly into proteomics and metabolomics, provided that the appropriate database files are available for evaluating pathway enrichment analysis. Finally, since Streamlit is based on a cloud platform, STAGEs can potentially work on both computer and mobile devices, providing users the option to share and analyze their gene expression data on different computing platforms.

The webtool is easy to maintain, and we intend to keep the gene lists from 2021 even if the gene sets continue to be updated over time. The benefit for using Streamlit for webtool development is that bioinformaticians can copy and manipulate the codes and run STAGEs locally to either add-on or exclude databases. The instructions for running STAGEs locally are detailed in GitHub (https://github.com/kuanrongchan/STAGES). Alternatively, users can directly upload their own gene sets onto the app for Enrichr or GSEA analysis. However, in the event where there are too many gene sets that significantly slow down the web-tool, we will send notifications within the app and GitHub, and ensure that the webtool is updated to the most current databases.

Currently, GSEA pre-rank analysis may take a long time to execute if large gene sets such as GOBP or Reactome datasets are used for pathway enrichment analysis. This is because the pathway analysis utilizes the full list of genes within the dataset, and multiple iterations are performed to improve consistency and reliability of the analysis. In addition, huge heatmaps and STRING queries with a large number of gene terms are usually not so easily readable. Presently, the webtool cannot perform batch correction, dimension reduction analysis and machine learning, and we have not evaluated the speed of data analysis if a big number of users are utilising the webtool. These limitations may be reconsidered in the next versions of STAGEs.

## Conclusion

In summary, we developed a publicly available, user-friendly and customisable web tool that allows transcriptomic data analysis. We believe this tool can assist non-coding users with their gene expression studies.

## Methods

### Creating vaccinomics database file

Peak vaccine responses against YF17D^2,24^, LAIV^25^, DVC-LVS^26^, MRKAd5/HIV^11^, rVSV-ZEBOV^27,28^, H5N1 + AS03^29^, MPSV4^18^, MCV4^18^, Hepatitis B adjuvanted with AS01B, AS01E, AS03, AS04 or Alum^30^, RTS,S/AS01/AS02^31^, Pneumovax23^32^ and influenza inactivated vaccines^33^ were used for making the .gmt Vaccinomics file (See Supplementary Table 1 for full details). At peak vaccine responses, fold-change of 1.3 and adjusted p-value (Benjamini–Hochberg Step-Up FDR-controlling procedure) < 0.05 was applied to filter for differentially expressed genes, which were subsequently used to annotate the vaccine gene sets in the database.

### Running STAGEs in web browser

STAGEs is available to everyone and the running instance of the app can be located at https://kuanrongchan-stages-stages-vpgh46.streamlitapp.com/. The documentation and instructions for use are available within the STAGEs app, and in this scientific publication.

### Running STAGEs locally

All codes, files, detailed instructions and the technical requirements are available at the GitHub repository (https://github.com/kuanrongchan/STAGES). Briefly, Streamlit and Python 3.7 (or later), together with several python packages (pandas >  = 1.3.4, numpy >  = 1.20.3) should be installed locally. A number of other requirements will have to be installed as well, where the requirements.txt file can be found in the GitHub repository. To install all requirements, users can type: *pip install -r requirements.txt.* Other files that should also be copied from our STAGEs repository include hgnc-symbol-check2.csv for the Gene Updater and demo_dataframe_corrected.csv for the demo dataset. For simplicity, users can also opt to download all required files as a ZIP file within the GitHub repository.

After specifying the directory and folder with the downloaded files in terminal using the change directory (cd) command, users can then simply type: *streamlit run stages.py*. This will generate a new tab with the STAGEs web tool appearing in the default browser. A docker image may also be built and run in a container with the Dockerfile within the repository with the instructions in the README file to facilitate reproducibility and convenience for users.

### Statement

All experiments and methods were performed in accordance with relevant guidelines and regulations.

## Supplementary Information

Supplementary Information.

## Data Availability

Codes that are used to generate STAGEs are hosted at https://github.com/kuanrongchan/STAGES. The Vaccinomics database file and demo dataset files are also located within the GitHub repository. This page can also be used to communicate any issues, queries or request features.
