# Supplementary material for: STAGEs: A web-based tool that integrates data visualization and pathway enrichment analysis for gene expression studies
Source: Sci Rep. 2023 May 2;13:7135. doi: 10.1038/s41598-023-34163-2 (PMC10153041; doi:10.1038/s41598-023-34163-2)
Supplement: Supplementary file 1 — Supplementary Information. [file 41598_2023_34163_MOESM1_ESM.docx]

**Supplementary Table 1: Datasets analysed to curate Vaccinomics database file**

|  | **Reference** | **Vaccine** | **Time** | **Description** |
| --- | --- | --- | --- | --- |
| 1 | Jue Hou et al., J Immunol, 2019 | Tiantan YF-17D | Day 7, 14 | 21 healthy subjects (age 25-42)  Direct transcriptomic analysis of PBMCs  Data deposited at GSE82152 |
| 2 | Querec TD et al., Nat Immunol, 2009 | YF-17D | Day 7 | 15 healthy subjects (age 18-45)  Direct transcriptomic analysis of PBMCs  Data deposited at GSE13486 |
| 3 | Nakaya HI et al., Nat Immunol, 2011 | FluMist® (LAIV) | Day 7 | 28 healthy subjects received LAIV (age 18-50)  Direct transcriptomic analysis of PBMCs  Data deposited at GSE29619 |
| 4 | Goll JB et al., Vaccines, 2020 | DVC-LVS | Day 7 | 10 healthy subjects (age 18-45).  Direct transcriptomic analysis of PBMCs  Data deposited at GSE149809 |
| 5 | Zak DE et al., PNAS, 2012 | MRKAd5/HIV | Day 1 | 7 seronegative subjects (age 20-50)  Direct transcriptomic analysis of PBMCs  Data deposited at GSE149809 |
| 6 | Santoro F et al., Vaccines, 2021 | rVSV-ZEBOV | Day 1 | 51 healthy subjects (age 18-65)  Direct transcriptomics analysis of whole blood  Data deposited at ZEN3974486 |
| 7 | Rechtien A et al., Cell Rep, 2017 | rVSV-ZEBOV | Day 1 | 18 healthy subjects (age 18-55)  Direct transcriptomics analysis of whole blood  Data deposited at GSE97590 |
| 8 | Wimmers F et al., Cell, 2021 | H5N1+AS03 | Day 1 | 34 healthy subjects (age 18-40) received H5N1+AS03  Direct transcriptomics analysis of PBMCs  Data deposited at GSE102012 |
| 9 | Li S et al., Nat Immunol, 2014 | Menomune® (MPSV4)  Menactra™ (MCV4) | Day 7 | 13 healthy subjects receive MPSV4, 17 receive MCV4 (age 18-45)  Direct transcriptomic analysis of PBMCs  Data deposited at GSE52245 |
| 10 | Laurane DM et al., Sci Transl. Med., 2020 | Adjuvanted hepatitis B (AS01B, AS01E, AS03, AS04, Alum) | Day 1 | 18 healthy subjects receive AS01B, 23 for AS01E, 28 for AS03, 22 for AS04, 21 for Alum, which are the different adjuvants for the HBs antigen.  Direct transcriptomic analysis on whole blood  Data deposited at GSE116975 |
| 11 | Vahey MT et al., J Infect Dis., 2010 | RTS,S/AS01 or RTS,S/AS02 | Day 1 | 39 healthy subjects, with 13 showing protection against *P. falciparum* (3D7 strain) challenge  Direct transcriptomic analysis of PBMCs  Data deposited at GSE18323 |
| 12 | Obermoser G et al., Immunity, 2013 | Pneumovax23 | Day 7 | 6 subjects receive Pneumovax23.  Direct transcriptomic analysis on whole blood  Data deposited at GSE30101 |
| 13 | Nakaya HI et al., Immunity, 2015 | Fluzone® (TIV)  (2010 – 2011) | Day 1 | 58 healthy subjects (age 20 - 86 years) receive TIV  Sampling at 0, 1, 3, 7, 14 days  Direct transcriptomic analysis of PBMCs  Data deposited at GSE74813 |
